# Novel Role for the Innate Immune Receptor Toll-Like Receptor 4 (TLR4) in the Regulation of the Wnt Signaling Pathway and Photoreceptor Apoptosis

**DOI:** 10.1371/journal.pone.0036560

**Published:** 2012-05-17

**Authors:** Hyun Yi, Amit K. Patel, Chhinder P. Sodhi, David J. Hackam, Abigail S. Hackam

**Affiliations:** 1 Bascom Palmer Eye Institute, University of Miami Miller School of Medicine, Miami, Florida, United States of America; 2 Department of Surgery (Pediatric), University of Pittsburgh, Pittsburgh, Pennsylvania, United States of America; Federal University of São Paulo, Brazil

## Abstract

Recent evidence has implicated innate immunity in regulating neuronal survival in the brain during stroke and other neurodegenerations. Photoreceptors are specialized light-detecting neurons in the retina that are essential for vision. In this study, we investigated the role of the innate immunity receptor TLR4 in photoreceptors. TLR4 activation by lipopolysaccharide (LPS) significantly reduced the survival of cultured mouse photoreceptors exposed to oxidative stress. With respect to mechanism, TLR4 suppressed Wnt signaling, decreased phosphorylation and activation of the Wnt receptor LRP6, and blocked the protective effect of the Wnt3a ligand. Paradoxically, TLR4 activation prior to oxidative injury protected photoreceptors, in a phenomenon known as preconditioning. Expression of TNFα and its receptors TNFR1 and TNFR2 decreased during preconditioning, and preconditioning was mimicked by TNFα antagonists, but was independent of Wnt signaling. Therefore, TLR4 is a novel regulator of photoreceptor survival that acts through the Wnt and TNFα pathways.

## Introduction

Toll-like receptors (TLRs) are essential mediators of innate and adaptive immunity in the defence against invading pathogens. Accordingly, TLRs are highly expressed on immune cells that have pathogen surveillance activity [1], [2]. However, the detection of TLRs on other cell types, such as neurons and glia, suggests additional physiological functions for TLRs. TLRs in the central nervous system (CNS) are activated by endogenous molecules released from injured cells that act as danger signals, known as damage-associated molecular patterns (DAMPs) [1], [3], [4], [5]. TLR4 in particular is increasingly being recognized as a modulator of neuronal survival in the brain during non-pathogen (sterile) injuries [6]. TLR4 is upregulated in many neurodegenerative diseases and neuronal injuries [7], [8] and also increases when neurons are exposed to toxic proteins and lipid peroxidation products [9]. Excessive activation of TLR4 and other TLRs induces expression of cytokines and pro-inflammatory molecules, resulting in further neuronal damage [10]. Indeed, induction of the TLR4 innate immunity pathway during oxidative and ischemic injuries promotes severe axonal and neuronal loss [9], [11], [12], [13], [14]. Furthermore, mice lacking TLR4 show reduced neuronal apoptosis and decreased pathology in the retina and brain [13], [14], which lends further support for a pathologic role of TLR4 in neuronal injury.

Paradoxically, low levels of TLR4 activation are believed to be beneficial to the CNS, and lead to a mild immune response, interferon production and reduced neuronal death. For example, low doses of LPS applied prior to CNS injury decreases neuronal damage during subsequent injury, in a phenomenon known as preconditioning [15], [16]. Therefore, precise regulation of TLR activity plays an important, yet poorly understood, role in neuronal injury and survival.

The Wnt pathway is an essential signaling cascade that regulates numerous processes in embryonic and adult tissues, including cellular proliferation, survival and differentiation. Our group and others recently demonstrated that Wnt signaling is increased during neuronal injury in the retina and is neuroprotective to retinal neurons and cell lines [17], [18], [19], [20], [21]. However, endogenous regulators of Wnt signaling are unknown. Interestingly, TLR4 was recently reported to down-regulate the Wnt pathway in enterocytes in the ileum of newborn mice [22], raising the possibility that TLR4 may regulate Wnt signaling and thereby influence photoreceptor survival.

Photoreceptors are light-sensing cells in the retina, which is the thin, multi-layer tissue at the back of the eye that is essential for vision. In the present study, we investigated the consequences of TLR4 activation on photoreceptor survival and tested whether TLR4 modulates the neuroprotective property of Wnt signaling. In summary, our findings show that TLR4 reduced photoreceptor survival in the presence of oxidative stress. Additionally, TLR4 suppressed Wnt-dependent protection of photoreceptors, and decreased phosphorylation of the Wnt pathway mediator LRP6 but not GSK3β. Furthermore, TLR4 activation prior to oxidative stress protected photoreceptors, and this preconditioning effect involved TNFα and was not dependent on Wnt signaling. Because damage and death of photoreceptors is a major cause of retinal degeneration diseases, our results implicate TLR4 in regulating photoreceptor death during retinal degeneration by interfering with the neuroprotective activity of Wnt signaling.

## Results

### TLR4 is Expressed in Muller Glia and Photoreceptors

Muller glia are the major radial glia type in the retina that provides trophic support to photoreceptors. Photoreceptor survival is influenced by proteins within the photoreceptors themselves as well as proteins secreted from adjacent Muller glia. We first examined whether TLR4 was expressed in these relevant cell types, using immunohistochemistry on dissociated Muller glia-photoreceptor co-cultures. The co-cultures are enriched (>99%) for Muller glia and photoreceptors, as previously described in [17], and offer the advantage of eliminating the contribution of other TLR4-responsive cells, such as microglia and astrocytes.

**Figure 1:**
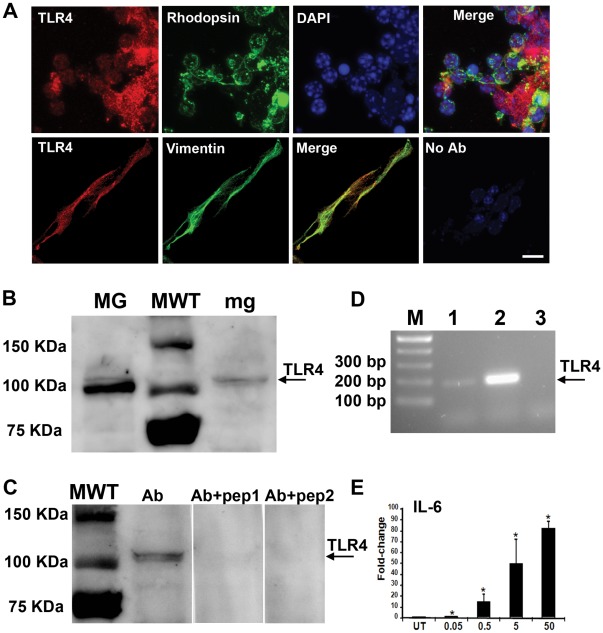
Expression of TLR4 in primary Muller glia-photoreceptor co-cultures. (A) TLR4 (red) is expressed in rhodopsin-positive photoreceptors (green). DAPI marks the nuclei. Detection of TLR4 and rhodopsin in the same photoreceptor cell is indicated by label overlap and is shown in the merged image. TLR4 (red) was also detected in vimentin-positive Muller glia (green). No antibody control indicates absence of non-specific immunostaining. Confocal microscopy, 63×. Scale bar is 20 µm. (B) Western blot showing detection of TLR4 protein (arrow) in whole cell lysates from the Muller glia cell line MIO-M1 (MG). The microglia cell line BV-2 was used as a positive control (mg). MWT, molecular weight marker, with sizes indicated on the left. (C) The specificity of the anti-TLR4 antibody was confirmed by preincubating with its peptide epitope. Detection of the TLR4 protein was evident with the antibody alone (Ab) but was mostly eliminated by 100 µg of the peptide antigen (Ab+pep1), and entirely eliminated by 200 µg (Ab+pep2). (D) TLR4 was detected by RT-PCR from primary cultures of Muller glia. Lane 1, diluted cDNA; lane 2, undiluted cDNA; lane 3, no template control. Lack of signal in the control indicates the specificity of the PCR reaction. (E) Dose-dependent induction of the TLR4 target gene IL-6 by LPS demonstrates that TLR4 is functional in the Muller glia cell line MIO-M1. IL-6 expression was measured by QPCR, and normalized to the house-keeping gene ARP. Mean ± SD, *p<0.05, n = 4, compared with untreated UT.

TLR4 was detected in both Muller glia and photoreceptors, as shown in Figure 1. Immunostaining for TLR4 overlapped with rhodopsin-positive photoreceptors and vimentin-positive Muller glia (Figure 1A). TLR4 expression in primary Muller glia culture and the Muller glia cell line MIO-M1 was confirmed by Western blotting (Figure 1B–C) and PCR (Figure 1D–E). The specificity of the anti-TLR4 antibody was indicated by the disappearance of the 100 kDa TLR4 band after preincubating the antibody with the peptide used to create the antisera (Figure 1C). Also, the TLR4 activator LPS induced a dose-dependent increase in the target gene IL6 in a Muller glia cell line, providing functional evidence of TLR4 activity in glia (Figure 1E).

**Figure 2:**
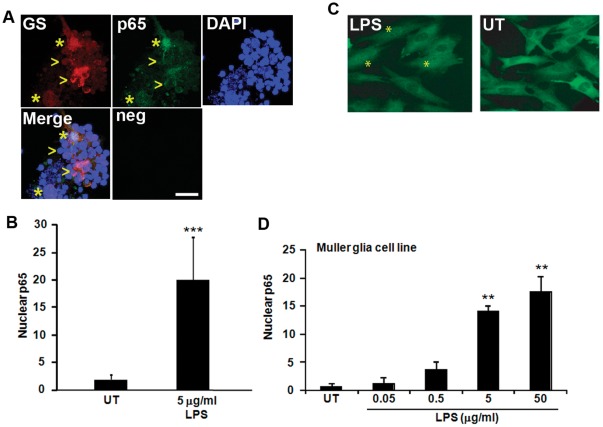
TLR4 is functional in primary Muller glia-photoreceptor co-cultures and a Muller glia cell line. (A) Nuclear translocation of the downstream TLR4 effector p65 was used as a marker of TLR4 activation. Muller glia-photoreceptor cultures treated with LPS (5 µg/ml) show nuclear localized p65 in Muller glia, as shown by p65 detection in the nuclei of cells costaining with the Muller glia marker glutamine synthetase (GS, asterisks). The arrowheads indicate GS-positive cells that contain cytoplasmic p65, indicating lack of TLR4 activation. Neg, immunostaining negative control. (B) Quantification of cells with p65 signal that overlapped with DAPI stained nuclei in glutamine synthetase-positive Muller glia. Mean ± SD, ***, p<0.001, n = 5. (C–D) MIO-M1 cells treated with LPS also show nuclear p65 (*) compared with untreated cells (UT). Dose-dependent nuclear translocation of p65 by LPS, indicating TLR4 activation in the Muller glia cell line. Mean ± SD, **, p<0.01, n = 5. Scale bar in (A) is 10 µm.

To further confirm that TLR4 is functional in the Muller glia-photoreceptor primary co-cultures, we quantified nuclear localization of p65/RELA, a downstream target of TLR4 through the NFkB pathway. Upon TLR4 activation, the NFkB/p65 complex is liberated from its inhibitor in the cytoplasm and translocates into the nucleus where it activates transcription. Nuclear p65 is often used as a marker of TLR4 activity [23]. The function of TLR4 in response to LPS treatment was examined in the Muller glia because Muller glia are a primary source of growth factors in the retina and have been shown to increase photoreceptor viability during injury through Wnt signaling [17]. The Muller glia-photoreceptor cultures showed a significant increase in nuclear localized p65 in glutamine synthetase-positive Muller glia after treatment with LPS (Figure 2A–B, p<0.001, n = 5). LPS also induced a dose-dependent increase in nuclear localization of p65 in the Muller glia cell line MIO-M1 (Figure 2C–D, p<0.01, n = 5). These data demonstrate that the primary cultures are an appropriate *in vitro* model to investigate the role of TRL4 in photoreceptors and Muller glia in response to LPS stimulation.

### Activation of TLR4 Decreases Photoreceptor Survival

The expression of TLR4 in Muller glia and photoreceptors led us to ask what is its potential role in photoreceptor survival. The Muller glia-photoreceptor primary co-cultures were used to test the effect of TLR4 activation on photoreceptor viability. The co-cultures reflect the response of only the two cell types of interest, Muller glia and photoreceptors, and have been used previously to investigate mechanisms of photoreceptor survival in many studies, particularly when fewer cell types and absence of systemic factors are preferred [24], [25]. The TLR4-responsive microglia cell type represents fewer than 0.005% of the cells in the culture, as detected by an antibody against the IBA-1 protein [17].

**Figure 3:**
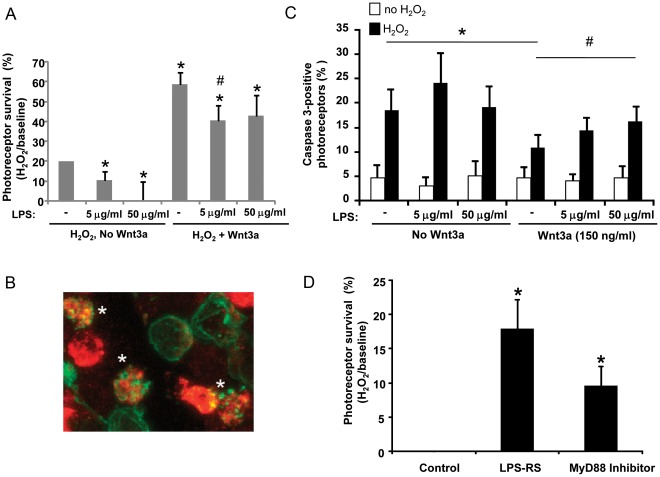
The TLR4 activator LPS decreases photoreceptor viability and reducesWnt-mediated protection from oxidative stress. (A) Muller glia-photoreceptor cultures were incubated for 24 hr in 0.4 mM H_2_O_2_ to induce oxidative stress, with and without recombinant Wnt3a (150 ng/ml) or the TLR4 activator LPS (5 and 50 µg/ml). Viability was measured by Cell Titer Blue. (A) LPS significantly reduced photoreceptor survival in the presence of H_2_O_2_ whereas Wnt3a is protective (*p<0.05 vs H_2_O_2_ alone). Furthermore, LPS reduced the protective effect of Wnt3a (#p<0.05 vs Wnt3a). Mean ± SEM, n = 4. (B) Confirmation that LPS blocks Wnt3a rescue of photoreceptors. Active caspase 3 (red) marks rhodopsin-positive photoreceptors (green) photoreceptors dying from H_2_O_2_. *, represents label overlap, indicating the proteins are detected in the same cell. Confocal microscopy, 63×, n = 4. (C) Quantification of overlap indicates that Wnt3a reduced the number of caspase-positive photoreceptors and LPS decreased Wnt3a protection. LPS is not toxic in the absence of H_2_O_2_. White bars, no H_2_O_2_; black bars, with H_2_O_2_. Mean ± SD, *p<0.05 no additions with H_2_O_2_ (n = 5) compared with Wnt3a (n = 4); #p<0.05 50 µg/ml LPS+Wnt3a (n = 5) compared with Wnt3a+ H_2_O_2_ (n = 4). (D) Retina cultures were incubated with LPS-RS (5 µg/mL) and the MyD88 inhibitor decoy peptide (0.5 µM) to block endogenous TLR4 activity, and cell viability in response to H_2_O_2_ injury was measured as above. Viability was measured by Cell Titer Blue and was normalized to the respective controls (PBS for LPS-RS, control peptide for MyD88 inhibitor). The inhibitors significantly increased viability. Mean ± SD, *p<0.05, n = 3.

H_2_O_2_, a commonly used inducer of oxidative stress, was used to injure the photoreceptors. Oxidative stress was chosen because it is a key contributor to photoreceptor death in vivo [26]. Previous studies with this experimental design demonstrated that photoreceptors were susceptible to H_2_O_2_ (99% of dead cells) and not Muller glia (1%), and showed that Wnt3a protected photoreceptors from H_2_O_2_-induced death [17]. Muller glia-photoreceptor co-cultures were incubated in 0.4 mM H_2_O_2_ in the presence or absence of recombinant Wnt3a and LPS. The prototypic ligand LPS was used to activate TLR4 in these experiments because the endogenous activators of TLR4 in the retina are unknown. As shown in Figure 3A, LPS significantly reduced photoreceptor survival in the presence of H_2_O_2_ whereas Wnt3a was protective (p<0.05 vs H_2_O_2_ alone, n = 4). Furthermore, the combination of LPS and Wnt3a with H_2_O_2_ had lower survival than Wnt3a with H_2_O_2_, indicating that LPS reduced the protective effect of Wnt3a (p<0.05 vs Wnt3a, n = 4). LPS is not toxic in the absence of H_2_O_2_, indicating a requirement for injury (see Figure 3C).

To confirm that LPS induced photoreceptor apoptosis, we quantified the number of cells in which we detected both active caspase 3 and the photoreceptor marker rhodopsin. Label overlap (*) of rhodopsin-positive photoreceptors (green) with active caspase 3 (red) marks photoreceptors dying from H_2_O_2_ (Figure 3B). No label overlap was observed with the Muller glia marker vimentin and active caspase 3, indicating resistance to H_2_O_2_ (not shown). Quantification of overlap indicates that Wnt3a reduced the percent of activated caspase 3-positive photoreceptors consistent with the viability assay above (compare no additions vs. Wnt3a, Figure 3C) (p<0.05, n = 5 for no additions and n = 4 for Wnt3a). Furthermore, LPS prevented Wnt3a-mediated protection and increased the percent of caspase-positive photoreceptors (compare Wnt3a vs. 50 µg/ml LPS+Wnt3a) (Figure 3C, p<0.05, n = 4 for Wnt3a and n = 5 for 50 µg/ml LPS+Wnt3a). LPS did not increase the amount of caspase-positive photoreceptors in the absence of H_2_O_2_ (Figure 3C).

Next, we investigated whether blocking endogenous TLR4 activity affects photoreceptor survival. LPS-RS is a potent antagonist of TLR4 that competes with LPS for binding to myeloid differentiation protein 2 (MD-2) [27], and the MyD88 homodimerization inhibitory peptide blocks TLR activity by acting as a decoy by binding to the MyD88 TIR domain. The Muller glia-photoreceptor cultures were injured by H_2_O_2_ in the presence of LPS-RS or MyD88 inhibitor peptide, and photoreceptor survival was measured. LPS was not included in these experiments to allow testing of the effects of endogenous activation of TLR4 on photoreceptor viability. Both LPS-RS and the MyD88 inhibitor significantly increased survival (Figure 3D, p<0.05, n = 3), indicating that blocking endogenous TLR4 activation protects against oxidative-stress induced cell death.

### Activation of TLR4 Decreases Neuroprotective Wnt Signaling

**Figure 4:**
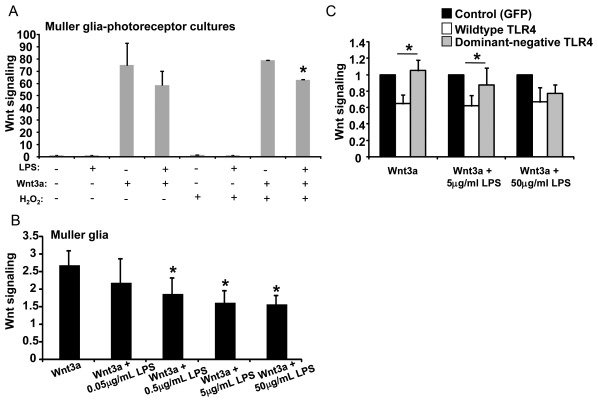
Potential mechanism of photoreceptor death: TLR4 suppresses neuroprotective Wnt signaling. The TLR4 activator LPS significantly decreased Wnt signaling in Muller glia-photoreceptor cultures (A) and the Muller glia cell line MIO-M1 (B–C), as measured by luciferase reporter assay. Normalization to cotransfected Renilla was used to account for the effect of LPS reducing cell number (*p<0.01, n = 4). (C) Wnt signaling is suppressed by wild-type TLR4 (white bars) but not dominant-negative (grey bars) (*p<0.05, n = 5). Luciferase activity was normalized to cotransfected Renilla luciferase, and is shown as the ratio of Wnt signaling in Wnt3a-treated cells to control-treated cells. Mean + SD is shown.

The viability results described above led us to investigate the relationship between TLR4 and Wnt signaling. Because Muller glia express both Wnt receptors and TLR4, and Wnt signaling protects photoreceptors against oxidative stress (Figure 3, [17]), we hypothesized that TLR4 activity contributes to photoreceptor death by suppressing the Wnt pathway in Muller glia during oxidative stress. To test the effect of TLR4 on Wnt signaling, Muller glia-photoreceptor co-cultures were cotreated with LPS and Wnt3a, or treated with Wnt3a alone. As shown in Fig. 4A, LPS resulted in a modest but significant decrease of Wnt signaling, by 21% (p<0.05, n = 3) in H_2_O_2_-injured Muller glia-photoreceptor co-cultures, as measured by Wnt signaling luciferase reporter assays.

Wnt3a increases Wnt signaling only in the Muller glia in the co-cultures because photoreceptors do not show active Wnt signaling pathways [17]. Therefore, a Muller glia cell line was used to further explore the relationship between TLR4 and Wnt signaling. The MIO-M1 Muller glia cell line offers the advantage of higher transfection efficiencies and larger cell numbers than the primary cultures. Treatment of MIO-M1 cells with increasing concentrations of LPS (0.5–50 µg/ml) resulted in dose-dependent suppression of Wnt3a-mediated Wnt signaling (Figure 4B, p<0.05, n = 3). To confirm that TLR4 mediated the LPS-dependent suppression of Wnt signaling, the cultures were transfected with wild-type TLR4 or a functionally inactive dominant-negative TLR4 mutant (dnTLR4). The dnTLR4 gene contains the inhibitory P712H mutation, is unresponsive to LPS and blocks endogenous LPS/TLR4 signaling [28], [29]. Transfection of dnTLR4 did not suppress Wnt signaling in the presence of LPS, and resulted in the same level of luciferase activity as the EGFP control gene (Figure 4C). In contrast, Wnt signaling was suppressed by wild-type TLR4 by 40% (Figure 4C, p<0.05, n = 5). Expression of TLR4 without added LPS also led to decreased Wnt signaling, consistent with TLR4 over-expression activating its pathway without exogenous ligands and providing additional evidence that TLR4 inhibits Wnt signaling.

Quantitation of the TLR4 target gene IL6 confirmed that dnTLR4 was unresponsive to LPS, that wild-type TLR4 was stimulated by LPS, and that wild-type TLR4 could also function independently of LPS addition (Figure S1, p<0.01, n = 4). Together, the data in Figures 3 and 4 suggest that the molecular basis for decreased viability of the Muller glia-photoreceptor primary cultures co-treated with LPS and Wnt3a is due to TLR4 decreasing Wnt signaling and reducing Wnt3a-mediated protection against H_2_O_2_.

### TLR4 Regulates the Wnt Pathway through LRP6

**Figure 5:**
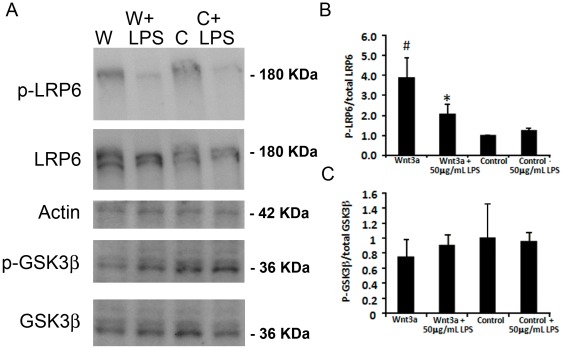
TLR4 decreases Wnt signaling at the level of the LRP6 receptor activation in Muller glia MIO-M1 cells. (A) Representative Western blots showing changes in phosphorylation status with Wnt signaling and LPS. Wnt3a conditioned media (W) increases the levels of phospho-Ser1490 LRP6 (p-LRP6) compared with control conditioned media (C). The addition of LPS reduces p-LRPS in Wnt3a treated cultures. In contrast, the amount of phospho-Ser9 in GSK3β is not affected by LPS. (B–C) Quantification of phospho-LRP6 and phospho-GSK3β. Twenty micrograms of total protein were loaded in each lane. The phosphorylated proteins were detected by phospho-specific antibodies and normalized to β-actin as a labeled control, and total LRP6 and GSK3β proteins were detected by antibodies that recognize both their respective phospho- and unphosphorylated forms, and normalized to β-actin. #, Wnt3a compared with control p<0.05, n = 5; *, Wnt3a compared with Wnt3a+LPS p<0.05, n = 5.

We next examined the mechanism by which TLR4 reduced Wnt3a-induced signaling. In the canonical Wnt pathway, Wnt ligands bind to Frizzled and LRP5/6 co-receptors at the plasma membrane, leading to recruitment of axin to the membrane and stabilization of the central mediator β-catenin [30], [31]. Phosphorylation of LRP6 at positions Ser1490, Thr1479 and Thr1493 is an important early activation step of the canonical Wnt pathway [32]. LRP6 phosphorylation at Ser1490 was measured in Muller glia MIO-M1 cells after Wnt3a addition in the presence or absence of 50 µg/ml LPS. Addition of Wnt3a lead to a 3.9-fold increase in phospho-LRP6 compared with control when tested after 1 hr, 2 hr and 4 hr, but not at 24 hr (Figure 5A–B, results for 1 hr are shown). However, LPS decreased phospho-LRP6 by approximately 50% (Figure 5A–B, p<0.05, n = 6). Therefore, TLR4 activation by LPS interrupts Wnt signaling at an early point in the pathway.

The phosphorylation status of the Wnt pathway intermediate GSK3β at position Ser 9 was also examined. GSK3β is a critical inhibitor of the Wnt pathway that plays a central role in the “destruction complex” that maintains low levels of β-catenin [31]. Thus, decreased phosphorylation of GSK3β is often associated with enhanced Wnt pathway activation. Muller glia MIO-M1 cells treated with Wnt3a showed a small reduction in GSK3β phosphorylation on Ser 9 compared with control, which did not reach statistical significance (Figure 5C). Co-treating the cells with Wnt3a and LPS also did not result in significant changes in GSK3β phosphorylation when tested after 1 hr, 2 hr and 4 hr (results for 1 hr shown). Therefore, TLR4 activation is unlikely to suppress Wnt signaling by regulating GSK3β activity through Ser9 phosphorylation. The luciferase assays and LRP6 analysis together demonstrate that activation of TLR4 suppresses Wnt signaling in Muller glia cells.

### Preconditioning by TLR4 Protects Photoreceptors

Preconditioning occurs when exposure to low levels of an otherwise harmful stimulus protects against subsequent exposure to toxic levels of the same or different stimulus [15], [16]. Importantly, preconditioning by one stimulus can often induce tolerance to other types of injury and preconditioning by LPS has been demonstrated to protect against ischemia in the CNS and other tissues, raising the possibility that LPS-dependent preconditioning may protect photoreceptors against oxidative stress [33].

**Figure 6:**
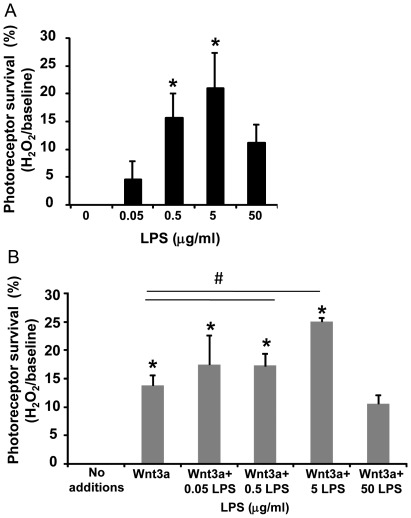
Preconditioning by LPS-induced TLR4 activation protects photoreceptors from oxidative stress. (A) Muller glia-photoreceptor cultures were incubated in LPS (0.05–50 µg/ml) two hrs before challenge with 0.4 mM H_2_O_2_. Significant protection was seen with 0.5 and 5 µg/ml LPS (*p<0.05 vs no LPS). Mean ± SD, n = 4. (B) Wnt3a (150 ng/ml) also protected photoreceptors (*p<0.05 compared with no additions). The combination of Wnt3a and 0.5 and 5 µg/ml LPS preconditioning had greater protection than Wnt3a alone (#p<0.05 vs Wnt3a). Mean ± SD, n = 4. The comparison between Wnt3a+5 µg/ml LPS vs Wnt3a+50 µg/ml LPS, Wnt3a+0.5 µg/ml LPS vs Wnt3a+5 µg/ml LPS, and Wnt3a+50 µg/ml LPS vs no additions, are also significant at p<0.05. Viability was measured by Cell Titer Blue.

To test whether TLR4 activation by LPS induces a preconditioning response in the Muller glia-photoreceptor co-cultures, we stimulated TLR4 by adding LPS 2 hours prior to exposure to oxidative stress, and photoreceptor viability was measured after an additional 24 hours. There was significant protection from cell death observed with 0.5 and 5 µg/ml LPS compared with cultures that did not receive LPS (Figure 6A, p<0.05, n = 4). Treatment with Wnt3a (150 ng/ml) also protected photoreceptors, as observed in Figure 3 and Yi et al. [17], and the combination of Wnt3a and LPS-induced preconditioning had greater protection than Wnt3a alone, suggesting an additive effect (Figure 6B, p<0.05, compared with Wnt3a, n = 4). Therefore, in contrast to the damaging effect of TLR4 activation *during* injury (Figure 3), preconditioning by TLR4 activation by LPS *prior* to an injury resulted in increased photoreceptor viability. Indeed, the importance of the timing of LPS treatment relative to injury is shown using the 5.0 µg/ml dose, which is toxic when LPS is cotreated with H_2_O_2_ but is protective when added 2 hr before H_2_O_2_. LPS-induced tolerance is usually a lower dose than the toxic dose [16], which is also shown in our results because preconditioning was significant with 0.5 µg/ml LPS whereas 5.0 µg/ml was required for toxicity to photoreceptors.

**Figure 7:**
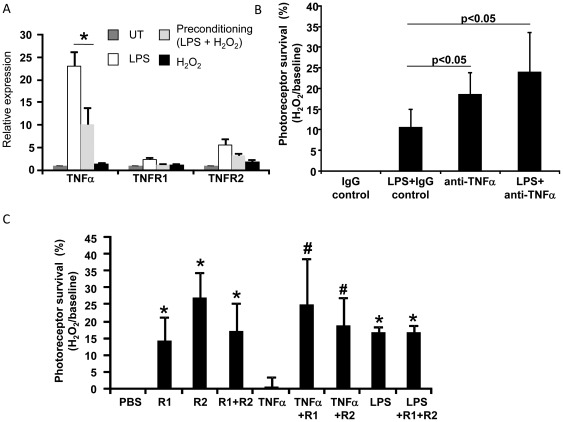
LPS-induced preconditioning involves TNFα signaling. (A) Primary Muller glia-photoreceptor cultures were treated with 5 µg/mL LPS 2 hr prior to 0.4 mM H_2_O_2_ and expression of TNFα and its receptors were analyzed by QPCR. The preconditioned cultures (LPS+H_2_O_2_) have lower expression of TNFα and the receptors TNFR1 and TNFR2 (mean ± SEM, *p<0.05, n = 4). (B) Muller glia-photoreceptor cultures were treated with 5 µg/ml anti-TNFα blocking antibody, or the IgG control, with or without LPS, 2 hr prior to H_2_O_2_ injury. Photoreceptor viability was measured and normalized to PBS treated cultures. The viability of photoreceptors treated with a combination of anti-TNFα antibody and LPS was over 2-fold higher than LPS cotreated with the IgG control (p<0.05, n = 6). Anti- TNFα antibody alone also showed photoreceptor protection, indicating that it substitutes for LPS. Viability was measured by Cell Titer Blue. (C) Muller glia-photoreceptor co-cultures were treated with 50 ng/ml of the TNFR1 and TNFR2 receptor inhibitors, with or without LPS, 2 hr prior to H_2_O_2_ injury. The TNFR1 and TNFR2 inhibitors protected the retina cultures, separately and in combination (*p<0.05, n = 6). Treating the cultures with TNFR1 and R2 inhibitors in combination with LPS did not enhance preconditioning more than LPS alone, suggesting a maximum level of protection was achieved by LPS. The addition of recombinant TNFα (5 ng/ml) resulted in photoreceptor viability equivalent to PBS control, which was reversed by TNFR1 and R2 inhibitors (#p<0.05, n = 6), indicating their effectiveness. Viability was measured by Cell Titer Blue andwas normalized to PBS treatments.

To investigate the mechanism of preconditioning, we first examined expression of candidate mediators downstream of TLR4 activation to determine whether there was a correlation between expression changes and increased photoreceptor survival. TNFα expression was significantly increased in the LPS-treated cultures compared with untreated cultures (Figure 7A), consistent with it being a downstream target of LPS. The expression of TNFα receptors TNFR1 and TNFR2, which are also targets of LPS, showed a mild, but not statistically significant, increase in the LPS-treated cultures (Figure 7A). In contrast, in preconditioned cultures (LPS+ H_2_O_2_), expression of TNFα was 2.3 fold lower than LPS-treated retinal cultures in the absence of H_2_O_2_ (p<0.05, n = 3). Similarly, the receptors TNFR1 and TNFR2 were 2-fold and 1.7-fold lower in the preconditioned cultures, respectively, compared with LPS treatment alone. Therefore, these data suggest that decreased TNFα signaling is associated with photoreceptor preconditioning to H_2_O_2_.

We next investigated whether TNFα signaling plays a role in LPS-induced preconditioning. If preconditioning requires reduced TNFα signaling, then suppressing the TNFα pathway further is expected to enhance the protective activity of LPS. First, an anti-TNFα blocking antibody was used to inhibit the activity of endogenous TNFα. As shown in Figure 7B, the viability of photoreceptors treated with a combination of anti-TNFα antibody and LPS was over 2-fold higher than LPS cotreated with the IgG control (Figure 7B, p<0.05, n = 6). The anti-TNFα antibody alone showed photoreceptor protection, indicating that it was able to substitute for LPS. Additionally, the viability of photoreceptors was significantly increased by inhibitors of the TNFR1 and TNFR2 receptors (50 ng/ml) in the absence of LPS (Figure 7C, p<0.05, n = 6). The protection by the combination of TNFR1 and TNFR2 inhibitors was equivalent to both added separately, indicating their protective effect was not additive. Treating the cultures with TNFR1 and TNFR1 inhibitors in combination with LPS did not enhance preconditioning more than LPS alone, suggesting a maximum level of protection was achieved by LPS (Figure 7C). The addition of recombinant TNFα (5 ng/ml) reduced photoreceptor viability to the level of the PBS control, which was reversed by TNFR1 and TNFR1 inhibitors (p<0.05, n = 6), indicating their effectiveness.

**Figure 8:**
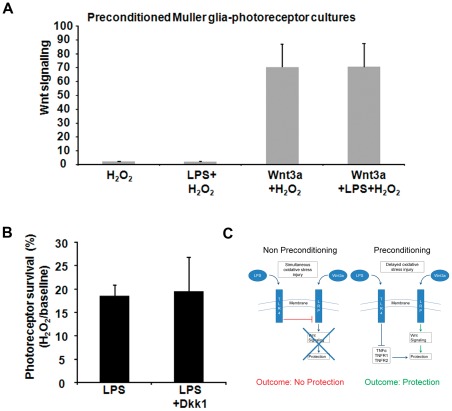
Preconditioning by LPS does not require canonical Wnt signaling. (A) Wnt signaling was measured in preconditioned Muller glia-photoreceptor cultures. The cultures were treated with LPS with or without Wnt3a, 2 hr prior to injury by H_2_O_2_ (0.4 mM). Wnt signaling was measured using a luciferase reporter assay. Wnt3a increased Wnt signaling (p<0.05, n = 5, compared with H_2_O_2_ only) but preconditioning with LPS did not induce Wnt signaling or change the level of Wnt3a-dependent Wnt signaling. Therefore, LPS-induced preconditioning is independent of the canonical Wnt pathway. (B) The Muller glia-photoreceptor cultures were incubated with LPS with or without the Wnt pathway inhibitor Dkk1 (200 ng/ml), followed by exposure to H_2_O_2_. The protection by LPS was not altered by Dkk1, indicating that endogenous Wnt pathway activation is not required. Mean ± SD, n = 5, compared with PBS treated. Viability was measured by Cell Titer Blue. (C) Proposed model of regulation of photoreceptor neuroprotection by cross-talk between the TLR4 and Wnt signaling pathways. (Left) During oxidative stress injury, TLR4 signaling suppresses Wnt signaling at the level of LRP6 receptor activation, leading to reduced Wnt3a-mediated photoreceptor protection. (Right) In the presence of LPS preconditioning prior to oxidative stress, activation of TLR4 reduces TNFα, leading to increased neuroprotection. TLR4 does not regulate the Wnt signaling pathway during preconditioning.

Wnt signaling reporter assays were used to determine whether preconditioning is associated with Wnt signaling. Cultures treated with recombinant Wnt3a showed Wnt signaling induction, as expected (Figure 8A, p<0.05, n = 5). However, Wnt signaling in LPS-induced preconditioned cultures (LPS+ H_2_O_2_) was not higher than H_2_O_2_ alone, and the preconditioned cultures in the presence of Wnt3a (Wnt3a+ LPS+ H_2_O_2_) was also not significantly different than non-preconditioned cultures (Wnt3a+H_2_O_2_) (Figure 8A), despite Wnt3a+LPS+ H_2_O_2_ having significantly greater viability than Wnt3a+ H_2_O_2_ (Figure 6B). Therefore, LPS-induced protective preconditioning does not depend on Wnt3a-induced survival pathways. This result contrasts with LPS-induced toxicity, which was associated with suppressed Wnt signaling (see Figure 4).

To test whether inhibiting endogenous Wnt signaling would reduce LPS-induced preconditioning, the Muller glia-photoreceptor co-cultures were incubated with LPS combined with 200 ng/ml of the Wnt pathway inhibitor Dkk1, followed by exposure to H_2_O_2_ to induce injury. As shown in Figure 8B, Dkk1 did not significantly change the viability of the cultures compared with LPS alone, confirming that preconditioning occurs without endogenous Wnt pathway activation. A proposed model describing the regulation of photoreceptor neuroprotection by cross-talk between TLR4 and Wnt signaling pathways, as derived from our experiments, is shown in Figure 8C.

### TLR4 Distribution in a Mouse Model of Retinal Degeneration

**Figure 9:**
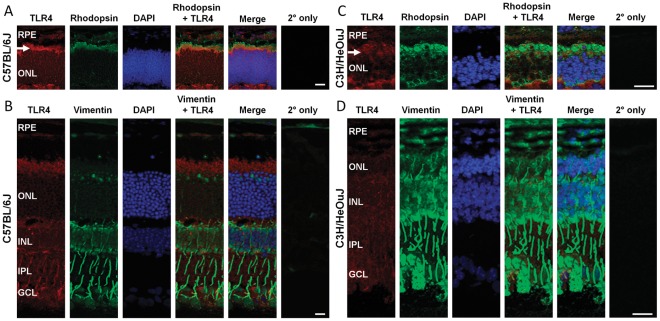
Localization of TLR4 in photoreceptors and Muller glia by immunodetection of TLR4, rhodopsin and vimentin in wild-type (C57Bl/6) and degenerating (C3H/HeOuJ) mouse retinas. (A,C) Both strains of mice were analyzed at post-natal day 14, which corresponds to the peak of rod photoreceptor degeneration in C3H/HeOuJ. TLR4 (red) is expressed in photoreceptors, shown by label overlap with rhodopsin (green, arrow) within the inner segments of C57 retinas, and in inner segments and outer nuclear layer (ONL) in C3H retinas. Sections stained with 2° antibody only (red, Alexa 546; green, Alexa 488) were used as controls for nonspecific staining. (B,D) TLR4 is expressed in Muller glia, as shown by label overlap of TLR4 (red) with the Muller glia protein vimentin (green). Expression of TLR4 within the Muller glia processes are clearly evident in the inner plexiform layer (IPL). Images in A–B are 10× magnification, and C–D are 20× magnification, using a Zeiss confocal microscope. DAPI was used to label the nuclei. RPE: Retinal pigmented epithelium, ONL: Outer nuclear layer, INL: Inner nuclear layer, IPL: Inner plexiform layer, GCL: Ganglion cell layer, Scale bar, A–C 25 µm, D 12 µm.

We next analyzed the expression pattern of TLR4 in retinas with photoreceptor degeneration to determine whether TLR4 is expressed in photoreceptors and Muller glia in vivo. The C57Bl/6 mouse was used as a wild-type control, an antibody against rhodopsin was used to identify rod photoreceptors, and an antibody that detects vimentin was used to identify Muller glia. In the C57Bl/6 retina, there was prominent TLR4 immunostaining in rhodopsin-positive photoreceptors, which was localized in the region corresponding to photoreceptor inner segments (arrows in Figure 9A). TLR4 immunodetection also overlapped with vimentin-positive cellular extensions, indicating TLR4 expression in Muller glia (Figure 9B). This expression pattern is consistent with our findings in the Muller glia-photoreceptor co-cultures (Figure 1). The specificity of the anti-TLR4 antibody was confirmed by lack of signal in TLR4 knock-out mice (Figure S2) and by lack of TLR4 immunodetection in the no primary antibody negative controls (Figure 9A–D). Signal overlap analysis is shown in Figure S3.

Next, the expression of TLR4 during photoreceptor death was analyzed using the C3H/HeOuJ mouse model of retinal degeneration (also known as *rd1*). The *rd1* strain carries the retinal degeneration allele *Pde6b^rd1^* and the wild-type *TLR4* allele [28], [34], [35] and exhibits rapid rod photoreceptor death. The retinas were analyzed at post-natal day 14, which corresponds to the peak of rod photoreceptor death. The thickness of the outer nuclear layer and the number of rows of photoreceptor nuclei in the outer nuclear layer was reduced in the *rd1* retina due to death of the rod photoreceptors (Figure 9C–D). TLR4 was located in the photoreceptor inner segments in the *rd1* retina, similar to wild-type retina, and was also prominently detected in the Muller glia (Figure 9C–D). Furthermore, the distribution of TLR4 in the photoreceptor layer changed during retinal degeneration and it became detectable in the outer nuclear layer, where the photoreceptor soma and nuclei are located (Figure 9C, Figure S2B). The new localization of TLR4 is similar to rhodopsin, which also becomes localized to the ONL, and may be due to a general mislocalization of proteins that occurs when the photoreceptor outer segments degenerate. TLR4 was not located in the outer nuclear layer in the age-matched wild-type mouse retina (Figure 9A, Figure S2A). Therefore, TLR4 is localized to photoreceptors and Muller glia in wild-type and degenerating mouse retinas, similar to its localization in the primary co-cultures, suggesting that TLR4 is a potential candidate regulator of photoreceptor death in vivo.

## Discussion

In this study, we showed that activation of the innate immunity TLR4 receptor has a dual role in regulating photoreceptor survival. TLR4 activation in the presence of oxidative stress reduced photoreceptor survival, whereas stimulation of TLR4 prior to injury increased photoreceptor survival, in a phenomenon known as preconditioning. The mechanism of TLR4 toxicity involved suppression of Wnt-dependent photoreceptor rescue, whereas TLR4-dependent preconditioning involved suppression of TNFα activity. Therefore, TLR4 is an important regulator of photoreceptor viability in culture, and may be an important element in the pathogenesis of retinal diseases where inflammation is a factor.

Our results are consistent with previous findings showing a toxic role for TLR4 in the CNS [9], [10], [11], [12], [13], [14]. Ko et al. (2011) also found that TLR4 activation, in this case by subcutaneous injection of heat killed *M. tuberculosis*, induced oxidative damage to photoreceptor outer segments, which was associated with elevated photoreceptor death and increased TNFα expression [36]. In contrast, LPS increased viability of the photoreceptor cell line 661 W when coapplied with injury induced by the nitrous oxide donor SNP [37]. Studies using animal models indicated that excessive TLR4 signaling and induction of innate immunity pathways contribute to further tissue damage by promoting axonal damage and neuronal death from oxidative and ischemic injuries [9], [11], [12], [13], [14]. In CNS damage models, mice lacking TLR4 have smaller infarct sizes, lower TNFα, IL6 and NFkB, and better outcomes in neurological and behavioral assays [7], [14]. Also, lack of TLR4 improved survival of retinal ganglion cells from axotomy-induced death [13]. Therefore, the majority of studies point to a pathologic activity of TLR4 in the brain and retina during neuronal injury.

Although LPS was used to activate TLR4 in this study, TLR4 in neurons and astrocytes is believed to be activated during CNS damage by DAMPs that are passively released from injured cells, such as hsp70 [38], hyaluronic acid [39] and high-mobility group protein box-1 (HMGB1) [40], [41], [42]. TLR4 activation leads to the release of TNFα, interleukin-1β, and other cytokines, which exacerbates the inflammatory response [43]. DAMPs also induce downstream pathways involved in regeneration [44] and neurite outgrowth [45], thereby stimulating tissue repair. Several endogenous TLR4 activators are upregulated during photoreceptor oxidative injury *in vivo*, such as HMGB1 [40], [46], suggesting that they may be a source of TLR4 activation during retinal disease.

Regarding a mechanism of TLR4-mediated toxicity, our study is the first example of an innate immunity receptor regulating the neuroprotective Wnt signaling pathway in the CNS. Because Wnt signaling induces growth factors in Muller glia [47], a potential mechanism for TLR4-induced photoreceptor death is by TLR4 activation suppressing Wnt signaling, leading to reduced growth factor expression and secretion, and increased photoreceptor death in response to oxidative stress. However, other mechanisms of TLR4 mediated death may also be involved that are independent from reduced Wnt signaling, including a direct effect of TLR4 on MAPK, NFkB and Jak1/Stat1 signaling pathways [48], [49]. TLR4 has also been shown to mediate neuronal toxicity through caspase 3, neuronal iNOS and phosphorylation of the stress kinases ERK1/2, JNK1/2 and p38 [13].

In support of our findings for an interaction between TLR4 and Wnt pathways, Sodhi et al. demonstrated that TLR4 activation decreased β-catenin levels in enterocytes in a mouse model of necrotizing endocolitis (NEC) [22]. Whereas we found that TLR4 suppressed Wnt signaling in Muller glia by reducing phosphorylation and therefore activation of LRP6, TLR4 acted further downstream in enterocytes by increasing GSK3β activity via regulation of AKT [22]. Reduced Wnt signaling is believed to be a factor in impaired enterocyte proliferation in NEC, and inhibiting TLR4 restored Wnt signaling and rescued the enterocyte phenotype. Interestingly, TLR4 activation in different cell types in the intestine can lead to opposite effects on enterocyte proliferation, possibly due to cell-specific effects, age of the animal and extent and duration of inflammation [22]. Also, LPS reduced nuclear β-catenin levels and Wnt luciferase reporter activity in an osteoblast cell line [50]. However, in Drosophila, Toll/NFkB signaling upregulated the β-catenin-independent Wnt family member WntD [51], possibly indicating species-specific and context-dependent effects.

Innate immunity in the retina is typically mediated by the immune-regulator cells microglia and astrocytes, although TLR4 is also active on non-immune cells, including RPE, Muller glia and retinal neurons (the present study, [52], [53]). Neurons throughout the CNS are able to regulate the immune response, enabling them to engage immune receptors in non-pathogen/stress response situations in order to maintain homeostasis and recover from injury. For example, in an ischemic stroke model, neurons were the first cell types to induce TLRs, followed by increased expression in the infiltrating microglia [7]. TLR4 localized in photoreceptors and Muller glia may permit a more direct and immediate response to genetic or environmental injury compared with activation in microglia, and may also engage different signaling pathways. TLR4 expression on photoreceptors has been demonstrated early in development [54] and by transcript analysis of laser microdissected photoreceptors [37]. TLRs, including TLR4, mediate RPE phagocytosis of photoreceptor outer segments [52]. TLR4 activation in microglia also contributes to photoreceptor survival, because Ko et al. found that TLR4 in microglia led to photoreceptor death after injection of heat killed *M. tuberculosis* [36]. Understanding how the TLR response in the different cells culminates in the overall retina response to injury will be important for determining therapeutic application of TLR pathways.

Muller glia are the principle supportive glia in the retina that are stimulated in retinal degenerations as part of the intrinsic tissue response to injury [55], [56]. Important insights into retinal diseases will be gained from understanding how the Muller glia-mediated inflammatory response in the retina is regulated by genetic injury and normal aging, and the role of endogenous regulator proteins in suppressing innate immunity and inflammation. Our findings also indicate that Muller glia may be cellular mediators of photoreceptor viability in response to TLR4, in addition to any possible direct effects of TLR4 activation on photoreceptor viability, which have yet to be determined. Activated Muller glia are believed to secrete growth factors that protect photoreceptors from further damage [57], [58] [47], [59]. Furthermore, Muller glia-microglia interactions may regulate the overall protection response [56], and Muller glia also suppress the immune system [60], which could be relevant to reducing further injury in the retina.

To our knowledge, our findings are the first to demonstrate LPS-induced photoreceptor preconditioning, raising the possibility that preconditioning by LPS may protect photoreceptors in vivo, as has been shown with preconditioning induced by bright light [61] and hypoxia/ischemia [62]. LPS injection also protected the inner retina (primarily retinal ganglion cells) from elevated pressure induced by ischemia-reperfusion injury, in an iNOS-dependent mechanism [15]. Although the physiological correlate of LPS-induced preconditioning is not yet known, it may indicate a role for TLR4 acting as a sensor of tissue injury by binding to molecules released from damaged cells, degradation of the extracellular matrix and changes in cellular redox state [1]. Injured neurons secrete TLR4 activating DAMP molecules that stimulate microglia and other cells, potentially Muller glia, and may induce neuroprotective cytokines and growth factors without engaging acute inflammation and neurotoxicity [63].

The mechanism of preconditioning by TLR4 activation is believed to be induction of a mild inflammatory response [15], [64], [65]. Our findings are consistent with this idea because TLR4 preconditioning in photoreceptor cultures involved lower TNFα levels than TLR4 activation in the absence of injury. Whether TNFα is acting directly on photoreceptors or indirectly through Muller glia remains to be established. TNFα plays dual roles in the CNS and is a major mediator of neuroinflammation and toxicity yet can also confer neuroprotection, depending on the specific receptor and adapter proteins engaged [66]. Indeed, LPS-induced preconditioning in a rat model of stroke required upregulated TNFα and neuroprotective molecules [67]–[68]. However, TNFα in the retina is primarily neurotoxic. TNFα in the vitreous is elevated during retinal degeneration and TNFα blockers protect photoreceptors from degeneration in a retinal detachment model [69], [70]. We also showed that preconditioning involved a distinct pathway from Wnt signaling-dependent protection. Similarly, Barandon et al. found that ischemic preconditioning in the mouse heart did not alter β-catenin levels [71]. However, Brandon et al did implicate GSK3β in preconditioning, suggesting that β-catenin independent/non-canonical Wnt signaling may be a factor in ischemia preconditioning protection in the heart [71]. Therefore, the involvement of non-canonical Wnt signaling in preconditioning in the retina is a possibility.

Our work suggests a model in which photoreceptor injury leads to activation of the innate immunity receptor TLR4, which increases photoreceptor death by inhibiting Wnt signaling in Muller glia. In contrast, TLR4-mediated preconditioning via TNFα suppression, prior to oxidative stress injury, is a novel mechanism of neuroprotection for photoreceptors (Figure 8C). Recent studies have linked dysregulation of the innate immune system with several common diseases of the retina, including age related macular degeneration (AMD) [72], [73] and glaucoma [74]. In AMD, dysfunctional RPE are believed to trigger an inflammatory response [75], but the specific contribution of innate immunity to photoreceptor death is unknown. TLR4 may be activated by signature molecules found in AMD retinas, including oxidized lipids, lipofuscin and components of protein deposits known as drusen [72], [76]. Once activated, TLR4 could contribute to the pathogenesis of AMD by inducing neurotoxicity to photoreceptors, similar to its activity elsewhere in the CNS.

TLR4 attracted considerable attention and controversy recently because coding and non-coding polymorphisms in the *TLR4* gene were associated with AMD [77], [78], although subsequent studies only confirmed a genetic association with non-coding SNPs and AMD [77]. Multiple TLRs are expressed in retina cell types involved in AMD, including RPE, macrophages, Muller glia, photoreceptors and endothelial cells [52], [53] but the activity of TLR4 has not been examined during AMD or other photoreceptor disease. Our data in the retinal cultures suggest that TLR4 activity in Muller glia and/or photoreceptors may contribute to photoreceptor death in AMD. TLR4 may be stimulated in AMD eyes by chronic low-level injury that releases DAMPs, oxidized lipids or other tissue damage. Therefore, regulating TLR4 activity, or the expression of endogenous damage response molecules to prevent TLR4 activation, may be a potential therapeutic target for retinal diseases such as AMD.

## Materials and Methods

### Cell Culture and Reagents

All procedures involving mice were performed in accordance with the ARVO Statement for the Use of Animals in Ophthalmic and Vision Research and were approved by the Animal Care and Use Committee at the University of Miami, protocol number 10-078. The mouse lines used were C57Bl/6 and C3H/HeOuJ (as known as *rd1*) from Jackson Laboratory (Bar Harbor, Maine) and TLR4 knock-out mice [79]. Primary Muller glia-photoreceptor co-cultures were prepared as described in [17]. Briefly, retinas from wild-type mice at post-natal day (P) 8 mice were dissociated in activated papain for 30 min at 37°C, mixed with Neurobasal medium containing 1× LoOvo plus DNAse I (Invitrogen, Carlsbad, CA) and pelleted by low-speed centrifugation. The cell pellet was washed in neurobasal-LoOvo medium without DNAse and plated in neurobasal medium containing L-glutamine, B27 and antibiotics onto poly-D-lysine/laminin coated 96-well dishes at a density of 2.5×10^5^ cells per well. Immunohistochemistry using antibodies recognizing cell-type specific marker proteins were used to confirm the purity of the cultures.

The human Muller glia cell line MIO-M1 was a gift from Dr. Astrid Limb [80] and the mouse microglia cell line BV2 [81] were generously provided by Dr. Venkata Kakulavarapu (University of Miami). Both cell lines were maintained in Dulbecco modified Eagle medium (DMEM) supplemented with 10% fetal bovine serum, 1% penicillin and streptomycin and 10 µg/ml L-glutamate. Recombinant Wnt3a and Dkk1 were obtained from R&D Systems (Manassas, VA), ultra-pure LPS *E. Coli* 0111:B4 and the TLR4 antagonist LPS-RS were from Invivogen (San Diego, CA), recombinant TNFα and soluble TNF Receptor Type I and Type II molecules to block the receptors were from Peprotech (Rocky Hill, NJ). Wnt3a conditioned media was prepared from mouse L-cells stably expressing Wnt3a (ATCC, Manassas VA), filtered and mixed with equal parts normal media for use. The control conditioned media was prepared from parental L-cells. The antibodies used in this study are listed in Table 1.

**Table 1:** Antibodies used in the study.

Protein	Company	Dilution
TLR4	Abcam Inc	1∶75 (IHC)
TLR4	Santa Cruz	1∶50 (IHC), 1∶1000 (W)
Iba1	Wako Chemicals	1∶100 (IHC)
Active caspase 3	Cell Signaling	1∶200 (IHC)
p65	Santa Cruz	1∶50 (IHC)
LRP6	Cell Signaling	1∶1000 (W)
p-LRP6	Cell Signaling	1∶1000 (W)
GSK3β	Cell Signaling	1∶1000 (W)
p-GSK3β	Cell Signaling	1∶1000 (W)
Glutamine synthetase	Sigma	1∶300 (IHC)
TNFα	ThermoScientific	1∶100 (IHC)
β-actin	sigma	1∶5000 (W)
Vimentin	sigma	1∶300 (IHC)
rhodopsin	Millipore	1∶300 (IHC)

IHC, immunohistochemistry; W, Western blot.

### Photoreceptor Viability Assays

Muller glia-photoreceptor co-cultures in triplicate wells were treated with or without H_2_O_2_ in the presence of PBS control, recombinant Wnt3a and/or LPS, for 24 hours. Viability was measured using the Cell Titer Blue reagent (Promega, Madison, WI), which was added for 1 hour at 37°C and was quantified using an ELISA plate reader. Average absorbance measured for media plus treatment was subtracted from each test sample. Each experiment was performed at least three times on different days. For the preconditioning studies, LPS was added 2 hours before the addition of H_2_O_2_ and Wnt3a and viability was measured as above. The two hour time-point was chosen because it is within the range typically used in published preconditioning studies in neuronal cultures, In some experiments, recombinant inhibitory molecules (Wnt3a, Dkk1, TNFα and TNF Receptor Type I and Type II, see previous paragraph) were added in addition to, or instead of, LPS, 2 hours before H_2_O_2_ treatment.

Caspase 3 activation was also used as a marker of cell death, using the anti-active caspase 3 antibody (Cell Signaling, Danvers, MA). The Muller glia-photoreceptor co-cultures were immunostained with anti–vimentin and anti–rhodopsin antibodies to identify caspase 3-positive cells, and counterstained with DAPI to label the nuclei. To determine the percentage of apoptotic cells per treatment, the total number of cells was counted, and apoptotic cells were quantified by counting green fluorescence caspase 3 staining that overlapped with vimentin or rhodopsin. The cultures were counted in a masked fashion.

### Quantitative PCR

Total RNA was extracted from cell pellets using Trizol reagent (Invitrogen), according to the manufacturer’s directions as previously described [82]. One microgram of RNA was treated with DNase (Ambion, Austin, TX) and cDNA was synthesized using Thermoscript (Invitrogen). QPCR was performed using the iCycler thermocycler (BioRad, Hercules, CA) with primers that were designed to be specific to the gene of interest and were separated by at least one intron within the gene. The primers were: *TNFR1*: sense: 5′ TGAGTGCGTCCCTTGCAGCCA 3′, antisense 5′ CGGGCCTCCACCGGGGATATC 3′; *TNFR2* sense 5′ CCTCGGACACCGTGTGTGCG 3′, antisense 5′ AAGGCGCAGTACCTGCCAGC 3′; *TNFα*: sense 5′ TCTTCTCATTCCTGCTTGTGG 3′, antisense 5′ CACTTGGTGGTTTGCTACGA 3′*; IL6*: sense 5′ CCAATTTCCAATGCTCTCCT 3′, antisense 5′ ACCACAGTGAGGAATGTCCA 3′. Each gene was amplified in triplicate per QPCR experiment, and each experiment was performed at least five times on independently prepared samples. Relative transcript levels of each gene were calculated using the delta-delta C_t_ method using the house-keeping gene ARP, as described, [83]. Standard PCR was performed using *Taq* polymerase (New England Biosciences) for *TLR4* using the primers: *TLR4* sense 5′ TTTATTCAGAGCCGTTGGTG 3′, antisense 5′ CAGAGGATTGTCCTCCCATT 3′.

### Western Blotting

The human Muller glia cell line MIO-M1 was treated with Wnt3a conditioned media or control conditioned media in the presence or absence of 50 µg/ml LPS. The cells were then harvested and lysed in buffer containing proteinase and phosphatase inhibitor cocktail (50 mM Tris, pH7.4, 150 mM NaCl, 1% NP40, 0.05% SDS). Twenty micrograms of total protein were resolved in 10% SDS–PAGE gels using Tris-glycine buffer and the proteins were then transferred onto polyvinylidene fluoride (PVDF) membranes and probed using the antibodies that detect total GSK3β, total LRP6, phospho-GSK3β at Ser9, phospho-LRP6 at Ser1490, and β-actin, followed by several washes, incubation with horseradish peroxidase (HRP)-conjugated secondary antibodies (Santa Cruz Biotechnology Inc, Santa Cruz CA), and incubation with enhanced chemiluminescence reagent (ECL-plus) (GE Amersham, Pistacataway NJ). The proteins were detected with a Chemidoc imager (BioRad) and the bands were quantified using NIH Image J. The values were normalized to β-actin to correct for loading differences, and the relative phosphorylation status was expressed as phospho-GSK3β/total GSK3β and phospho-LRP6/total LRP6. The source of antibodies and dilutions are listed in Table 1.

An antigen blocking experiment was performed to confirm the specificity of the anti-TLR4 antibody. Briefly, 0.5 ml of antibody was preincubated with 100 µg and 200 µg of its peptide epitope (Santa Cruz Biotechnology Inc.) for 1 hr at room temperature. Identical fractions of MIO-M1 cell lysates were loaded into adjacent wells of an SDS-PAGE gel, transferred onto a PVDF membrane, and the membrane was cut into strips. The strips were incubated with anti-TLR4 antibody or the antibody-peptide mixes, washed, incubated in secondary antibody, and then assembled together for imaging, as described above.

### Wnt Signaling Activity Luciferase Assays

Primary Muller glia-photoreceptor co-cultures and cell lines were co-transfected with a 4∶1 ratio of the TOP-FLASH luciferase reporter plasmid (a generous gift from Dr. R. Moon, HHMI, University of Washington) and a Renilla luciferase plasmid (Promega) using a nucleofector electroporator (Lonza, Wakersville). Wnt signaling was induced by the addition of Wnt3a at 24 hr post-transfection. The cells were harvested after 24 hr into Reporter lysis buffer (Promega, Madison WI) [84], according to the manufacturer’s directions. Luciferase activity was measured in a Lumistar Galaxy luminometer (BMG Labtech Inc, Cary NC) and normalized to Renilla activity. Wnt signaling activity is expressed as firefly luciferase units/Renilla luciferase units. The assays were performed in triplicate wells in at least four independent experiments.

### Immunohistochemistry

Eyes were rinsed in PBS, fixed in 4% paraformaldehyde, incubated in increasing sucrose concentrations (5%–20%) then embedded in OCT and flash-frozen, as described [84]. Sections were cut at 10 µm thickness. Immunostaining of the Muller glia-photoreceptor co-cultures was performed on cultures plated onto glass chamber slides (Lab-Tek), as described in [17]. The retinas and retinal culture slides were blocked in non-immune serum from the species of the secondary antibody and incubated with primary antibody overnight at 4°C, washed in PBS, and then incubated with secondary antibody. The sections were counterstained with DAPI, mounted in a solution of glycerol/PBS and were viewed using a fluorescent microscope (Zeiss Axiovert 200), equipped with a 20×/NA 0.70 (HC Plan-Apochromat; Leica) objective lens, or a confocal microscope (Leica TCS SP5, Leica Microsystems), equipped with a 63×/NA 1.4 oil (Plan-Apochromat; Leica) objective lens. Image acquisition and analysis software was Axiovision LE (Carl Zeiss) using a cooled charge-coupled device camera or the Leica Application Suite (Leica), respectively. All sections were imaged at room temperature (20–22°C). Photographic and microscopic settings were kept constant for comparisons between antibody and control staining. The images were processed for publication using Photoshop (Adobe) with only minimal adjustment of brightness or contrast that was applied to the entire figure, including the positive and negative controls.

### Statistical Analysis

Student’s *t*-test or two-way analysis of variance (ANOVA) were used for statistical analyses using GraphPad Prism. A p-value <0.05 was considered significant.

## Supporting Information

Figure S1 **IL-6 expression analysis of wild-type and dominant-negative TLR4.** IL-6 values obtained by QPCR were normalized to the housekeeping gene ARP, and then to the GFP transfection control (*p<0.01, n = 4). Mean ± SD is shown. White bars, wild-type TLR4, grey bars, dominant-negative TLR4.
(TIF)

Figure S2 **Analysis of TLR4 expression in TLR4 knock-out (TLR4 KO) retinas.** Absence of TLR4 detection in Muller glia and photoreceptors indicates specificity of the anti-TLR4 antibody. Artifactual separation of the photoreceptor outersegments is denoted by (∧). Images are at 20× magnification using a Zeiss confocal microscope. DAPI was used to label the nuclei. RPE: Retinal pigmented epithelium, ONL: Outer nuclear layer, INL: Inner nuclear layer, IPL: Inner plexiform layer, GCL: Ganglion cell layer. Scale bar, A–C 25 µm, D 12 µm.
(TIF)

Figure S3 **Analysis of label overlap of TLR4 and rhodopsin and TLR4 and vimentin.** Confocal microscopy imaging software was used to identify regions of label overlap of TLR4 and the photoreceptor marker protein rhodopsin (A–D) and the Muller glia marker protein vimentin (E–H). The merged images (see also Fig. 9 for the images presented separately) were analyzed and regions of label overlap are indicated in white. Images are at 10× magnification for A–C and 20× for D, using a Zeiss confocal microscope. DAPI was used to label the nuclei. RPE: Retinal pigmented epithelium, ONL: Outer nuclear layer, INL: Inner nuclear layer, IPL: Inner plexiform layer, GCL: Ganglion cell layer, Scale bar, A–C 25 µm, D 12 µm.
(TIF)
