# Supplementary figures and images for: Novel Role for the Innate Immune Receptor Toll-Like Receptor 4 (TLR4) in the Regulation of the Wnt Signaling Pathway and Photoreceptor Apoptosis
Source: PLoS One. 2012 May 17;7(5):e36560. doi: 10.1371/journal.pone.0036560 (PMC3355158; doi:10.1371/journal.pone.0036560)

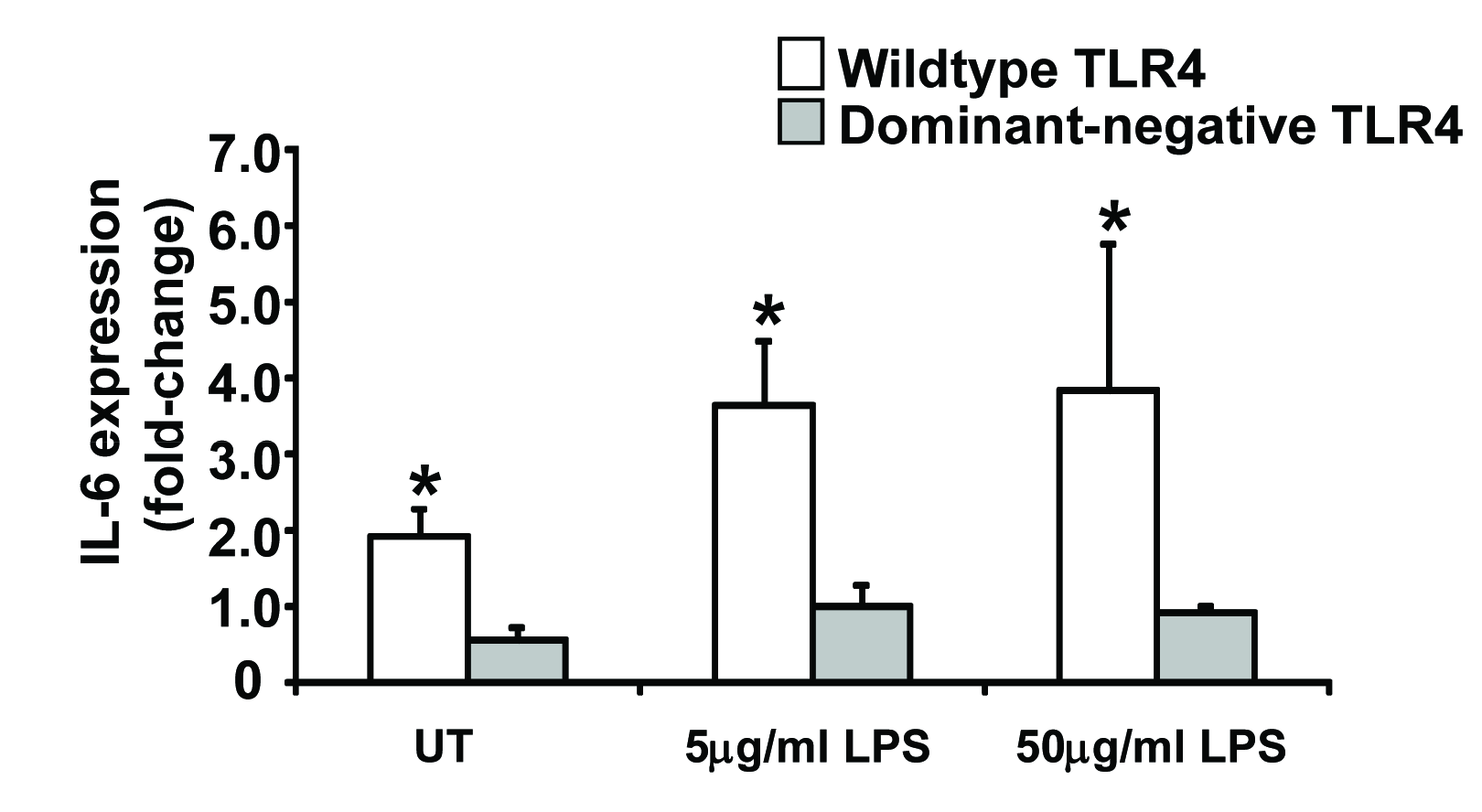

Supplement: Figure S1 — IL-6 expression analysis of wild-type and dominant-negative TLR4. IL-6 values obtained by QPCR were normalized to the housekeeping gene ARP, and then to the GFP transfection control (*p<0.01, n = 4). Mean ± SD is shown. White bars, wild-type TLR4, grey bars, dominant-negative TLR4. (TIF) [file pone.0036560.s001.tif]

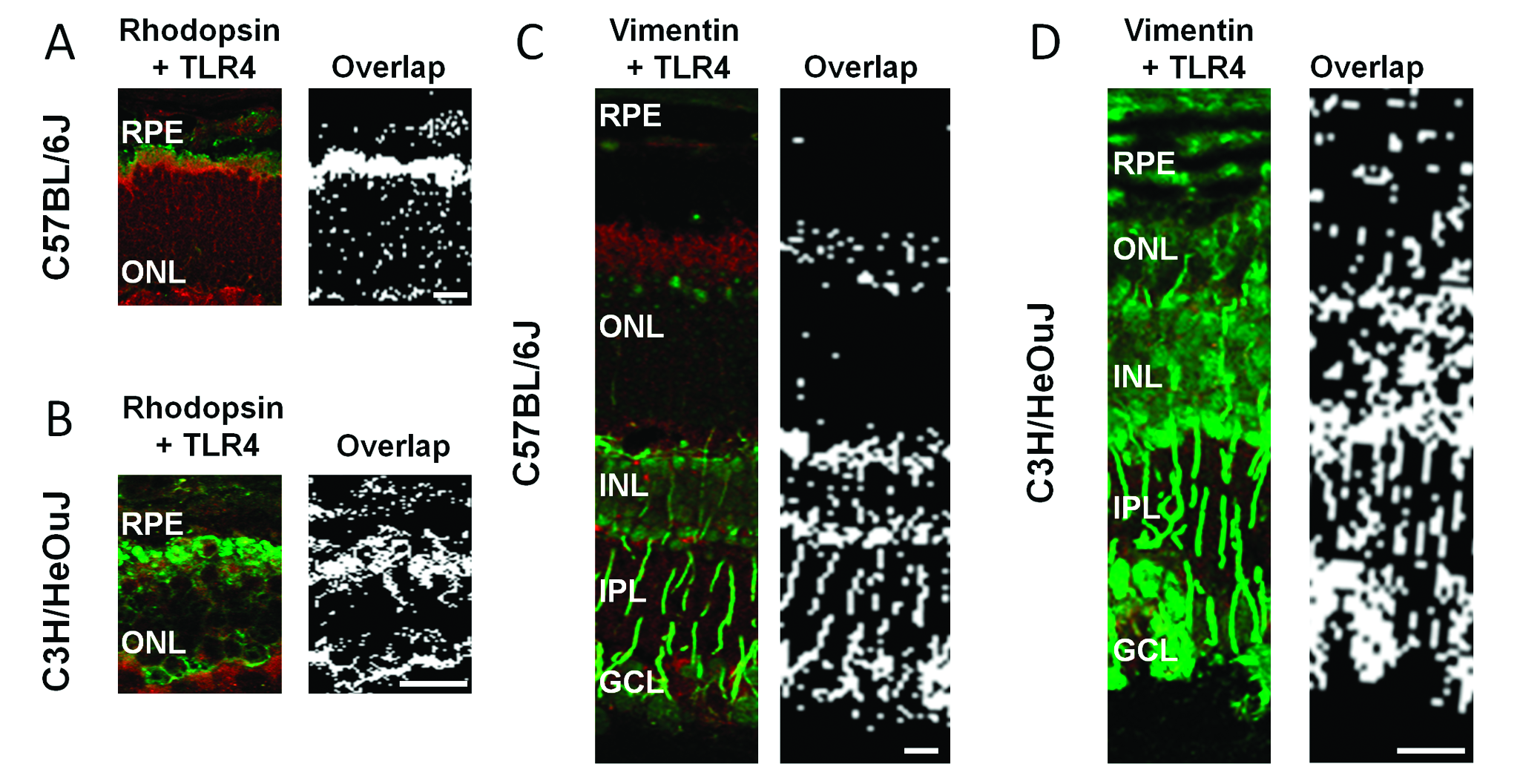

Supplement: Figure S2 — Analysis of TLR4 expression in TLR4 knock-out (TLR4 KO) retinas. Absence of TLR4 detection in Muller glia and photoreceptors indicates specificity of the anti-TLR4 antibody. Artifactual separation of the photoreceptor outersegments is denoted by (∧). Images are at 20× magnification using a Zeiss confocal microscope. DAPI was used to label the nuclei. RPE: Retinal pigmented epithelium, ONL: Outer nuclear layer, INL: Inner nuclear layer, IPL: Inner plexiform layer, GCL: Ganglion cell layer. Scale bar, A–C 25 µm, D 12 µm. (TIF) [file pone.0036560.s002.tif]

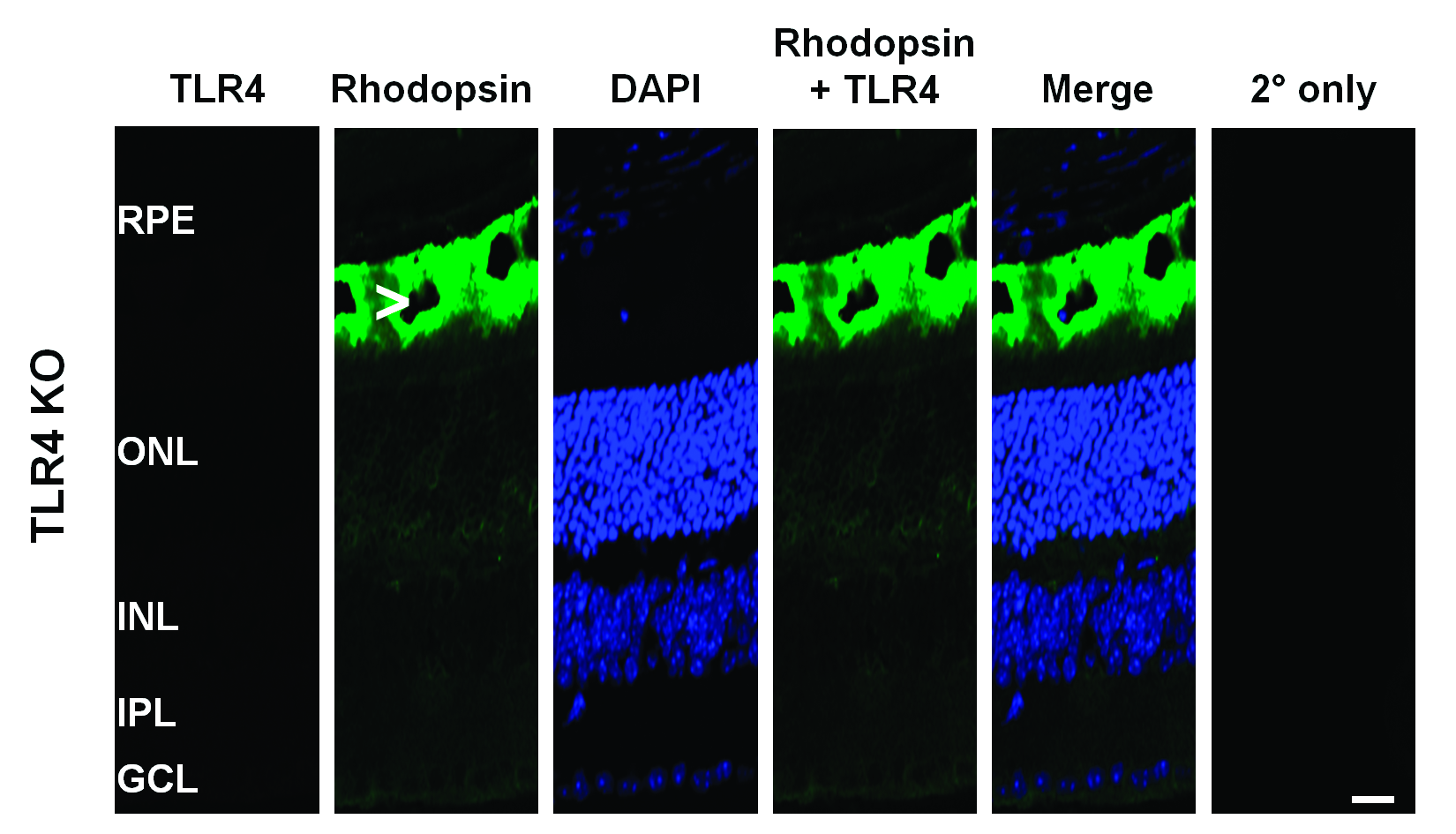

Supplement: Figure S3 — Analysis of label overlap of TLR4 and rhodopsin and TLR4 and vimentin. Confocal microscopy imaging software was used to identify regions of label overlap of TLR4 and the photoreceptor marker protein rhodopsin (A–D) and the Muller glia marker protein vimentin (E–H). The merged images (see also Fig. 9 for the images presented separately) were analyzed and regions of label overlap are indicated in white. Images are at 10× magnification for A–C and 20× for D, using a Zeiss confocal microscope. DAPI was used to label the nuclei. RPE: Retinal pigmented epithelium, ONL: Outer nuclear layer, INL: Inner nuclear layer, IPL: Inner plexiform layer, GCL: Ganglion cell layer, Scale bar, A–C 25 µm, D 12 µm. (TIF) [file pone.0036560.s003.tif]
